# Cone‐Beam Computed Tomography–Derived Alveolar Anatomy for Immediate Implant Planning: Thickness, Concavity, and Angulation Patterns: A Comprehensive Evaluation

**DOI:** 10.1155/ijod/9240109

**Published:** 2026-09-17

**Authors:** Lina Khayyal, Abeer Alhadidi, Omar Al-Karadsheh, Momen Atieh, Ahmad A. Hamdan

**Affiliations:** ^1^ School of Dentistry, University of Jordan, Amman, Jordan, ju.edu.jo; ^2^ Department of Oral and Maxillofacial Surgery, Oral Medicine, and Periodontology, School of Dentistry, University of Jordan, Amman, Jordan, ju.edu.jo; ^3^ Department of Oral and Maxillofacial Pathology, Radiology and Medicine, New York University College of Dentistry, New York, USA, nyu.edu; ^4^ Sir John Walsh Research Institute, Faculty of Dentistry, University of Otago, Dunedin, New Zealand, otago.ac.nz; ^5^ Hamdan Bin Mohammed College of Dental Medicine, Mohammed Bin Rashid University of Medicine and Health Sciences, Dubai, UAE, mbruniversity.ac.ae

**Keywords:** buccal bone, cone-beam computed tomography, dental implant, maxillary bone, tooth angulation

## Abstract

**Objectives:**

This study aimed to evaluate the buccal and palatal bone thickness, sagittal angulation, and the buccal bone concavity angle of maxillary anterior teeth using cone beam computed tomography (CBCT).

**Materials and Methods:**

This cross‐sectional study included patients ≥18 years with periodontally healthy maxillary anterior teeth (cementoenamel junction (CEJ)–bone crest (BC) ≤3 mm). Scans with artifacts, restorations, implants or pathological bone loss were excluded. Buccal and palatal bone thicknesses were measured at coronal, midroot, and apical levels, along with CEJ–BC distance, sagittal angulation, and buccal bone concavity angle. Data were analyzed using descriptive and parametric statistics, with Pearson correlation analysis.

**Results:**

A total of 112 CBCT scans (502 maxillary anterior teeth) were analyzed from 33 men and 79 women (mean age 31.10 years). Of 672 teeth examined, 170 were excluded mainly due to crestal/periapical bone loss, restorations, or imaging artifacts. Mean facial bone thickness was 1.05 ± 0.31 mm, while palatal bone thickness was 4.07 ± 3.07 mm. Mean CEJ–BC distance ranged from 1.65 ± 0.56 to 1.72 ± 0.56 mm. Mean sagittal angulation was 8.70° ± 5.09° for central incisors, 7.86° ± 5.15° for lateral incisors, and 12.23° ± 4.3° for canines. Buccal concavity angles averaged 154° ± 14.6°, 152° ± 9.33°, and 158° ± 7.7° for central incisors, lateral incisors, and canines, respectively. Significant correlations were observed between sagittal angulation and both buccal and palatal bone thicknesses, as well as between concavity angle and selected bone thickness measurements. Sensitivity analysis accounting for within‐patient clustering confirmed the robustness of the sagittal angulation correlations, whereas most correlations involving the concavity angle did not remain significant after clustering adjustment.

**Conclusion:**

The facial bone plate of the anterior maxilla is thin (<1.5 mm), with maxillary anterior teeth frequently positioned buccally within a curved labial plate. As an observational anatomical study, these findings would help in highlighting the importance of individualized CBCT‐based evaluation in treatment planning, particularly when considering augmentation needs and implant positioning in the esthetic zone.

## 1. Introduction

Dental implants have become the standard of care for replacing missing teeth, demonstrating high long‐term success and survival rates [1]. Achieving optimal outcomes requires prosthetically driven, three‐dimensional implant placement that respects the anatomical configuration of each site in the oral cavity [2]. In this context, the anterior maxilla represents a challenge due to its complex anatomical configuration and high esthetic demands. In this region, the cortical plate and alveolar bone merge closely around the anterior teeth, often with minimal cancellous bone present [3]. Moreover, the facial bone wall surrounding the buccally positioned roots of anterior teeth tends to be very thin [4] and mainly composed of bundle bone, which is a tooth‐dependent bone that undergoes substantial resorption upon tooth removal. Such resorption alters the anatomical relationships within the maxillary process, emphasizing the importance of careful preoperative assessment when planning dental implant placement.

Several studies have used cone‐beam computed tomography (CBCT) to evaluate facial bone dimensions in the esthetic zone, including buccal and palatal bone thickness [3, 5–7], the distance from the cementoenamel junction (CEJ) to the facial bone crest (BC) [7–9], buccal concavity along the labial plate [9–11], and the sagittal angulation between teeth and alveolar bone [12, 13]. However, few studies have examined potential relationships among these parameters to understand how they collectively influence implant placement and the risk of cortical perforation [14–16]. Therefore, this retrospective study aimed to assess the sagittal angulation and buccal bone concavity angle of maxillary central and lateral incisors and canines and to investigate their correlations with facial and palatal bone thickness, as well as CEJ‐facial BC distance. By evaluating these relationships, the study aims to improve our understanding of anatomical factors that influence implant positioning and the potential risk of cortical perforation in the esthetic zone, supporting the need for individualized CBCT‐based treatment planning.

## 2. Materials and Methods

### 2.1. Study Design and Data Collection

This retrospective cross‐sectional morphometric study was conducted and reported in accordance with STROBE guidelines, and it was approved by the Scientific Committee and Deanship of Academic Research at the University of Jordan (UJ) and the Institutional Review Board of Jordan University Hospital (IRB#221000370/2021‐2022).

### 2.2. Sample Size

A convenience sampling approach was used. CBCT data were obtained from the Oral Radiology Department database at Jordan University Hospital for Jordanian patients who attended the dentistry department between January 2015 and July 2022. CBCT scans were originally acquired for various diagnostic purposes, including implant planning, third molar assessment, and orthodontic evaluation. More than 1000 CBCT scans were screened (patient selection flowchart is available as a supporting file, Supporting Information 1: Figure S1). Patient age and gender were recorded, while patient identifiers were removed to ensure confidentiality. All CBCT datasets were stored in Digital Imaging and Communications in Medicine (DICOM) format and imported into Carestream Kodak Dental Imaging Software for analysis.

### 2.3. Inclusion Criteria

CBCT scans were included if they met the following criteria: (1) patients aged ≥18 years; (2) presence of bilateral permanent maxillary anterior teeth; and (3) normal maxillary bone levels, defined as crest bone level of ≤3 mm apical to the CEJ.

### 2.4. Exclusion Criteria

CBCT scans were excluded if orthodontic appliances, scatter or motion artifacts, implants, or metallic restorations were present in the region of interest. Individual teeth were excluded if they showed radiographic evidence of crestal or periapical bone loss, tooth surface loss, malalignment (e.g., rotation), full‐coverage restorations or veneers, incisal restorations, endodontic treatment, or root resorption.

### 2.5. Data Measurements

**Figure 1:**
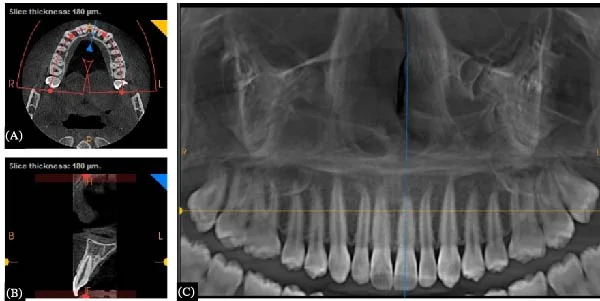
Some steps of the calibration: (A) the axial section of maxillary arch; (B) panoramic view of the maxilla with a vertical line runs along the long axis of the tooth; and (C) the sagittal section with the least slice thickness.

All measurements were performed by a single calibrated examiner following standardized training to ensure consistent navigation and measurement protocol. Sagittal sections were obtained by aligning the cursor in the panoramic view at the center of each maxillary anterior tooth, parallel to the long axis of the tooth. Measurements were performed using the thinnest slice thickness (0.2 mm) at 1:1 magnification (Figure 1).

To assess intraexaminer reliability, ten CBCT scans were re‐evaluated after a 2‐week interval. The intraclass correlation coefficient (ICC) demonstrated excellent agreement (ICC = 0.994; 95% confidence interval (CI): 0.992–0.994; *p*  < 0.001).

### 2.6. Sagittal Angulation and Buccal Concavity Angle

**Figure 2:**
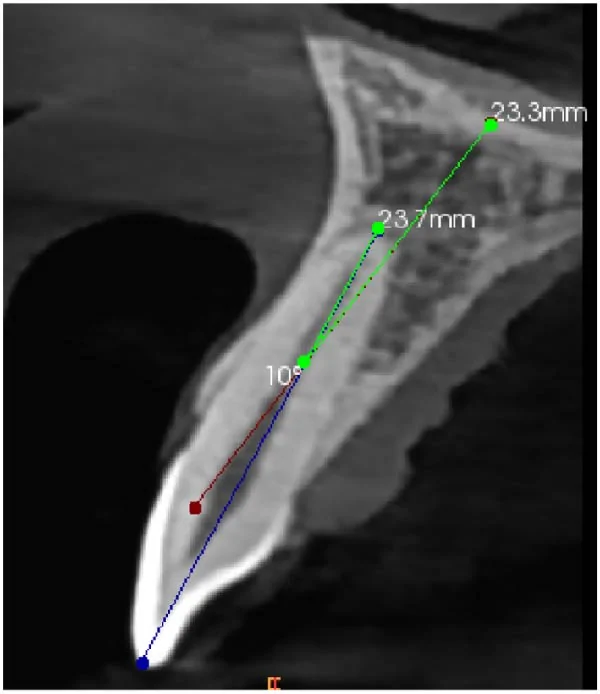
The sagittal angulation of the maxillary central incisor formed between the long axis of the tooth and the long axis of the alveolar ridge.

The sagittal angulation was measured as the angle between:

1. The long axis of the tooth (line connecting incisal edge/cusp tip to the root apex), and
2. The long axis of the corresponding alveolar bone (line bisecting the alveolar ridge buccolingually) (Figure 2).

**Figure 3:**
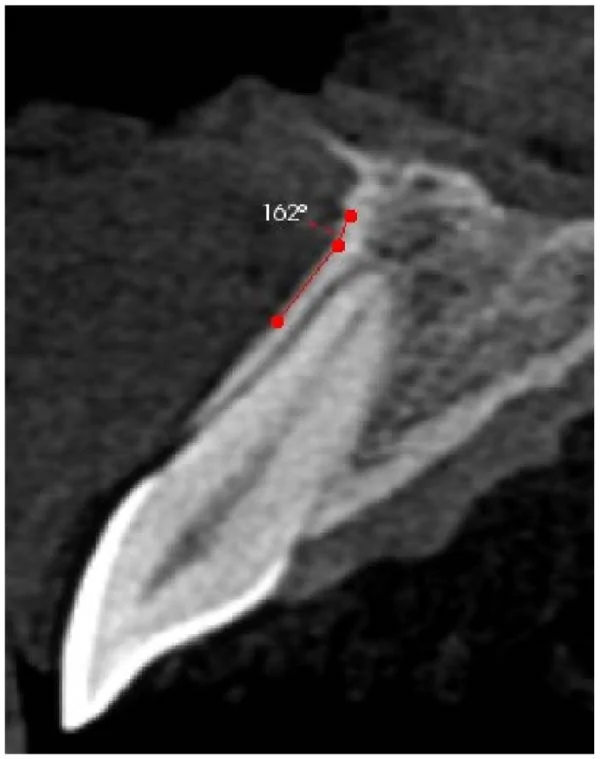
The buccal bone concavity angle formed between two tangent lines along the inclination of the facial bone plate.

The buccal bone concavity angle was defined as the angle formed by two tangent lines drawn along the inclination of the facial bone plate (Figure 3).

To evaluate correlations between sagittal angulation, buccal concavity angle, and other anatomical parameters, buccal and palatal bone thickness, as well as the distance between the facial alveolar bone crest and the CEJ, were also assessed.

### 2.7. Bone Thickness Measurements

Facial and palatal bone thicknesses were measured perpendicular to the long axis of the tooth at three standardized levels:

1. Coronal level: Within the first 3 mm apical to the BC, at the widest measurable point.
2. Midroot level: At the midpoint between the CEJ and root apex.
3. Apical level: At the level of the root apex.

**Figure 4:**
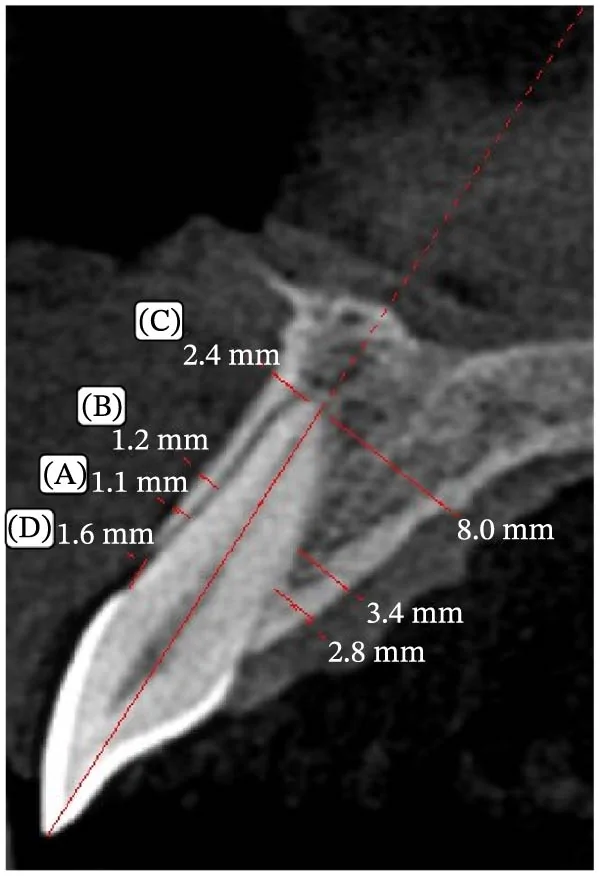
A sagittal section of the maxillary central incisor illustrating the following measurements: (A) facial bone thickness at coronal level; (B) facial bone thickness at midroot level; (C) facial bone thickness at apical level; and (D) distance of CEJ–BC.

The long axis of each tooth was defined as the line connecting the root apex to the incisal edge or cusp tip (Figure 4A–C).

### 2.8. CEJ–BC Distance

The CEJ–BC distance was measured as the linear distance between the most coronal point of the facial alveolar BC and the CEJ (Figure 4D).

### 2.9. Statistical Analysis

Statistical analysis was performed using the Statistical Package for the Social Sciences (SPSS) for Windows (version 26; SPSS Inc., Chicago, IL, USA). Data normality was assessed using the Kolmogorov–Smirnov test, and homogeneity of variances was evaluated using Levene’s test. Differences between the left and right sides of the maxilla were assessed using the paired sample *t*‐test, while gender‐based comparisons were performed using the independent *t*‐test. Pearson correlation coefficient (R) was used to evaluate correlations between sagittal angulation, facial and palatal bone thickness, CEJ–BC distance, and buccal bone concavity angle. Post hoc multiple comparisons were adjusted using the Holm–Bonferroni method to control for type I error. This correction was applied to the pairwise post hoc comparisons following the analysis of variance of sagittal angulation across buccal concavity‐angle groups; given the exploratory, hypothesis‐generating nature of the correlation analyses, correlation coefficients are reported with nominal (unadjusted) *p*‐values. Because multiple teeth from the same patient were included, gender comparisons and correlation analyses were additionally reassessed using generalized estimating equations (GEE) with an exchangeable working correlation structure, clustering observations by patient and using robust (sandwich) standard errors; this sensitivity analysis was performed to verify whether conclusions from the primary analysis were robust to within‐patient clustering. Data were reported as mean ± standard deviation (SD). Statistical significance was set at *p* < 0.05.

## 3. Results

A total of 112 CBCT scans for patients (79 females and 33 males) with a mean age of 31.1 years (range: 18–71 years) were included. In total, 502 of 672 maxillary anterior teeth were analyzed, including 177 central incisors, 183 lateral incisors, and 142 canines. The remaining 170 teeth were excluded due to different reasons such as apical root resorption, tooth surface loss, presence of incisal restorations, motion or metal artifacts, and/or radiographic signs of crestal bone loss.

### 3.1. Sagittal Angulation of Maxillary Anterior Teeth

Table 1 presents the mean sagittal angulation between the long axis of each tooth and the long axis of the corresponding alveolar bone. Maxillary canines demonstrated the greatest sagittal angulation (12.23° ± 4.27°), followed by central incisors (8.70° ± 5.09°) and lateral incisors (7.86° ± 5.15°) (Table 2).

**Table 1:** Mean values and standard deviations (SD) of each variable according to tooth and gender (mm; angles: degree).

Distribution		Central incisors				Lateral incisors				Canines			
		Mean ± SD	Women	Men	*p*‐Value	Mean ± SD	Women	Men	*p*–Value	Mean ± SD	Women	Men	*p*‐Value
Facial wall	Coronal	1.05 ± 0.25	1.06 ± 0.25	1.03 ± 0.27	0.627	1.20 ± 0.46	1.21 ± 0.50	1.16 ± 0.39	0.443	1.11 ± 0.36	1.14 ± 0.35	1.03 ± 0.42	0.152
	Midroot	0.77 ± 0.37	0.77 ± 0.34	0.80 ± 0.45	0.691	0.69 ± 0.64	0.72 ± 0.66	0.61 ± 0.59	0.277	0.55 ± 0.53	0.48 ± 0.51	0.79 ± 0.56	0.003 ^∗^
	Apical	1.33 ± 0.76	1.03 ± 0.76	1.41 ± 0.53	0.388	1.58 ± 1.13	1.43 ± 1.11	1.91 ± 1.14	0.008 ^∗^	1.18 ± 0.98	1.05 ± 0.92	1.60 ± 1.06	0.004 ^∗^
Palatal wall	Coronal	1.93 ± 0.57	1.83 ± 0.53	2.17 ± 0.59	~0.001 ^∗^	1.54 ± 0.61	1.42 ± 0.57	1.82 ± 0.60	~0.001 ^∗^	1.50 ± 0.53	1.39 ± 0.45	1.87 ± 0.64	~0.001 ^∗^
	Midroot	3.18 ± 1.07	2.93 ± 0.95	3.77 ± 1.14	~0.001 ^∗^	2.52 ± 1.17	2.21 ± 1.01	3.22 ± 1.21	~0.001 ^∗^	3.48 ± 1.56	3.15 ± 1.26	4.55 ± 1.93	~0.001 ^∗^
	Apical	7.59 ± 2.13	7.12 ± 1.92	8.70 ± 2.22	~0.001 ^∗^	5.98 ± 2.02	5.56 ± 1.93	6.90 ± 1.96	~0.001 ^∗^	9.35 ± 3.04	8.85 ± 2.60	10.92 ± 3.76	~0.001 ^∗^
CEJ–BC		1.66 ± 0.55	1.57 ± 0.52	1.87 ± 0.57	0.001 ^∗^	1.72 ± 0.56	1.70 ± 0.57	1.77 ± 0.53	0.482	1.65 ± 0.56	1.51 ± 0.51	2.09 ± 0.50	~0.001 ^∗^
Sagittal angulation		8.70 ± 5.09	7.83 ± 4.70	10.73 ± 5.45	~0.001 ^∗^	7.86 ± 5.15	7.85 ± 5.20	7.87 ± 5.0	0.968	12.23 ± 4.27	12.0 ± 4.30	13.0 ± 4.22	0.269
Buccal bone concavity angle		154.0 ± 14.6	154.1 ± 13.62	154.2 ± 17.0	0.973	152.5 ± 9.33	151.84 ± 9.80	154.1 ± 8.12	0.936	158.1 ± 7.7	157.5 ± 7.85	160 ± 7.22	0.103

*Note:* CEJ–BC: distance between cementoenamel junction and bone crest; thickness in mm; CEJ–BC distance in mm; angles in degree.

^∗^Significant at *p*  < 0.05.

**Table 2:** Differences between the mean values and standard deviations of CEJ–BC distance, sagittal angulation, and buccal bone concavity angle based on tooth type.

	Central incisor vs. Lateral incisors		*p*‐Value	Lateral incisor vs. Canine		*p*‐Value	Canine vs. Central incisor		*p*‐Value
	Central incisor	Lateral incisor		Lateral incisor	Canine		Canine	Central incisor	
CEJ–BC	1.65 ± 0.54	1.68 ± 0.57	0.417	1.70 ± 0.57	1.65 ± 0.56	0.392	1.65 ± 0.56	1.62 ± 0.53	0.583
Sagittal angulation	8.87 ± 5.14	7.68 ± 5.0	0.007 ^∗^	7.76 ± 4.73	12.36 ± 4.20	~0.001 ^∗^	12.31 ± 9.15	8.65 ± 5.2	~0.001 ^∗^
Buccal bone concavity angle	155 ± 14.3	152.7 ± 9.4	0.061	152.5 ± 9.40	158.4 ± 7.70	~0.001 ^∗^	158.0 ± 4.15	154.7 ± 5.18	0.023 ^∗^


*Note:* CEJ–BC: distance between cementoenamel junction and bone crest; thickness in mm; CEJ–BC distance in mm; angles in degree.

^∗^Significant at *p*  < 0.05.

Sagittal angulation was ≤ 10° at 66.1% of central incisors, 69.5% of lateral incisors, and 35.9% of canines. Angulation between 11° and 20° was observed in 31.6%, 29.5%, and 61.3%, respectively, while 21°–30° was observed in 2.3%, 1.1%, and 2.8%, respectively.

No significant differences were found between the right and left sides (Table 3). Male patients exhibited significantly greater sagittal angulation in central incisors (*p*  < 0.001), while no gender‐related differences were observed for lateral incisors or canines (Table 1).

**Table 3:** Mean values and standard deviations of each variable on both the right and left sides.

Distribution		Central incisors			Lateral incisors			Canines		
		Right	Left	*p*‐Value	Right	Left	*p*‐Value	Right	Left	*p*‐Value
Facial wall	Coronal	1.07 ± 0.28	1.01 ± 0.22	0.011 ^∗^	1.18 ± 0.50	1.15 ± 0.41	0.359	1.07 ± 0.32	1.09 ± 0.35	0.662
	Midroot	0.81 ± 0.36	0.72 ± 0.39	0.040 ^∗^	0.67 ± 0.65	0.64 ± 0.58	0.570	0.51 ± 0.49	0.53 ± 0.52	0.809
	Apical	1.40 ± 0.79	1.32 ± 0.74	0.363	1.54 ± 1.10	1.70 ± 1.20	0.086	1.11 ± 0.97	1.23 ± 1.07	0.461
Palatal wall	Coronal	1.94 ± 0.52	1.90 ± 0.60	0.543	1.53 ± 0.60	1.60 ± 0.62	0.274	1.51 ± 0.63	1.50 ± 0.48	0.919
	Midroot	3.23 ± 1.15	3.15 ± 1.04	0.526	2.55 ± 1.28	2.56 ± 1.15	0.914	3.60 ± 1.75	3.50 ± 1.43	0.576
	Apical	7.64 ± 2.23	7.64 ± 2.07	0.980	6.11 ± 2.11	5.93 ± 2.08	0.212	9.45 ± 3.10	9.72 ± 2.90	0.374
CEJ–BC		1.63 ± 0.54	1.67 ± 0.57	0.336	1.70 ± 0.56	1.75 ± 0.56	0.370	1.63 ± 0.58	1.66 ± 0.55	0.614
Sagittal angulation		9.02 ± 4.95	9.24 ± 5.11	0.644	7.85 ± 5.12	8.07 ± 5.39	0.061	12.8 ± 5.00	12.0 ± 4.11	0.144
Buccal bone concavity angle		153.1 ± 16.50	155.1 ± 15.60	0.220	151.7 ± 9.80	154.1 ± 8.80	0.026 ^∗^	158.4 ± 6.71	158.2 ± 7.97	0.851

*Note:* CEJ–BC: distance between cementoenamel junction and bone crest; thickness in mm. CEJ–BC distance in mm; angles in degree.

^∗^Significant at *p* < 0.05.

### 3.2. Buccal Bone Concavity Angle of Maxillary Anterior Teeth

The buccal bone concavity angle was highest at maxillary canines (158.1° ± 7.7°), indicating a less concave buccal plate compared with central incisors (154.0° ± 14.6°) and lateral incisors (152.5° ± 9.33°). No significant differences were observed between genders (Table 1). A statistically significant difference between right and left sides was observed only for lateral incisors (*p* = 0.026), with left lateral incisors showing slightly greater mean concavity angles than right lateral incisors (Table 3).

### 3.3. Facial and Palatal Bone Thicknesses of Maxillary Anterior Teeth

Facial and palatal bone thicknesses varied significantly according to tooth type and root level.

The overall mean palatal bone thickness was 4.24 ± 2.81 mm for central incisors, 3.35 ± 2.36 mm for lateral incisors, and 4.78 ± 3.88 mm for canines (Table 4). Mean facial bone thickness measured 1.05 ± 0.56 mm coronally, 0.67 ± 0.27 mm at the midroot level, and 1.37 ± 0.93 mm apically. Palatal bone thickness increased progressively from coronal to apical levels, measuring 1.67 ± 0.61 mm coronally, 3.03 ± 1.33 mm at midroot, and 7.50 ± 2.74 mm apically (Table 4). Palatal bone thickness was significantly greater than facial bone thickness at all measurement levels across all tooth types. Lateral incisors demonstrated slightly greater facial bone thickness compared with central incisors and canines, while presenting the lowest palatal bone thickness among the anterior teeth.

**Table 4:** Differences between the mean values and standard deviations of the facial and palatal bone thicknesses of maxillary anterior teeth.

	Coronal				Midroot			Apical			Overall mean of bone thickness	
	Facial		Palatal	*p*‐Value	Facial	Palatal	*p*‐Value	Facial	Palatal	*p*‐Value	Facial	Palatal
Central incisors	1.05 ± 0.25		1.93 ± 0.57	~0.001 ^∗^	0.77 ± 0.37	3.18 ± 1.07	~0.001 ^∗^	1.33 ± 0.76	7.59 ± 0.21	~0.001 ^∗^	1.05 ± 0.26	4.23 ± 0.53
Lateral incisors	1.20 ± 0.46		1.54 ± 0.61	~0.001 ^∗^	0.69 ± 0.64	2.52 ± 1.2	~0.001 ^∗^	1.58 ± 1.13	5.98 ± 2.02	~0.001 ^∗^	1.16 ± 0.61	3.23 ± 1.30
Canines	1.11 ± 0.36		1.50 ± 0.53	~0.001 ^∗^	0.55 ± 0.53	3.48 ± 1.6	~0.001 ^∗^	1.18 ± 1.00	9.35 ± 3.64	~0.001 ^∗^	0.95 ± 0.47	4.65 ± 5.30
Overall mean	1.12 ± 0.25		1.67 ± 0.32	—	0.67 ± 0.27	3.03 ± 1.7	—	1.37 ± 0.93	7.49 ± 5.70	—	1.05 ± 0.31	4.03 ± 3.07

*Note:* Thickness in mm.

^∗^Significant at *p*  < 0.05.

### 3.4. Distribution of Facial Bone Thickness

At the coronal level, 36.5% of maxillary anterior teeth exhibited facial bone thickness < 1 mm, 59.9% measured between 1 and 2 mm, and only 3.6% showed thickness ≥ 2 mm.

At the midroot level, 28.4% of teeth showed absence of facial bone, 44.7% showed thickness < 1 mm, 24.8% measured between 1 and 2 mm, and 2.1% showed thickness ≥ 2 mm.

At the apical level, 18% of teeth showed absence of facial bone, 17% showed thickness < 1 mm, 40% measured between 1 and 2 mm, and 25% showed thickness ≥ 2 mm.

### 3.5. Distribution of Palatal Bone Thickness

At the coronal level, 9.5% of teeth showed palatal bone thickness <1 mm, 61.3% measured between 1 and 2mm, and 29.2% showed thickness ≥2 mm.

At the midroot level, 2% showed thickness <1 mm, 18.7% measured between 1 and 2 mm, and 79.3% showed thickness ≥2 mm.

At the apical level, no teeth showed palatal bone thickness <1 mm, 1.2% measured between 1 and 2 mm, and 98.8% showed thickness ≥2 mm.

### 3.6. Distribution of Facial Bone Thickness in All Teeth

At the coronal level:

36.5% had <1 mm, 59.9% were 1–2 mm, 3.6% were ≥2 mm

At the midroot level:

28.4% had no facial bone, 44.7% had <1 mm, 24.8% had 1–2 mm, 2.1% had ≥2 mm

At the apical level:

18% had no bone, 17% had <1 mm, 40% had 1–2 mm, 25% had ≥2 mm.

### 3.7. Distribution of Palatal Bone Thickness

At the coronal level:

• 9.5% had <1 mm
• 61.3% had 1–2 mm
• 29.2% had ≥2 mm

At the midroot level:

• 2% had <1 mm
• 18.7% had 1–2 mm
• 79.3% had ≥2 mm

At the apical level:

• 0% had <1 mm
• 1.2% had 1–2 mm
• 98.8% had ≥2 mm

Comparison between different measurements in males and females is presented in Table 1. Males showed significantly greater facial bone thickness in lateral incisors at the apical level (men: 1.91 ± 1.14 mm; women: 1.43 ± 1.11 mm; *p* = 0.008) and in canines at midroot level (men: 0.79 ± 0.56 mm; women: 0.48 ± 0.51 mm, *p* = 0.003) as well as at apical level (men: 1.60 ± 1.06 mm; women: 1.05 ± 0.92 mm; *p* = 0.004), while there was no statistically significant difference at the other sites. For palatal bone thickness, Males had significantly greater palatal bone thickness at all levels across all tooth types (*p*  < 0.001) (*p* ~ 0.001).

No statistical differences were found between the means of the right and left facial bone thickness except that of the central incisors, in which the right central incisors had a mean value greater than the left central incisors at coronal (*p* = 0.011), and midroot levels (*p* = 0.040). In addition, no significant differences were found between the means of the right and left mean values of the palatal bone thickness at all levels for all tooth types (Table 3).

### 3.8. The Measurements of Distance Between CEJ and Facial BC of Maxillary Anterior Teeth

The CEJ–BC means remained consistent across tooth types: 1.66 ± 0.55 mm (centrals), 1.72 ± 0.56 mm (laterals), and 1.65 ± 0.56 mm (canines) (Table 1).

When means of the right and left sides were compared, no significant differences were found (Table 3). Males showed statistically significant higher means of CEJ–BC distance than females at central incisors (*p*  ~ 0.001) and canines (p ~ 0.001) (Table 1).

### 3.9. Correlation Analyses

The correlation analyses examined relationships among sagittal angulation, buccal concavity angle, facial/palatal bone thickness, and CEJ–BC distance.

### 3.10. Correlations With Sagittal Angulation

#### 3.10.1. The Facial Bone Dimension

The thickness of the facial bone wall of maxillary anterior teeth had no significant correlation coronally with the sagittal angle, while negative correlations were found at midroot level of central incisors (*R* = −0.177, *p* = 0.018) and canines (*R* = −0.231, *p* = 0.006) and at apical levels of central incisors (*R* = −0.426, *p* ~ 0.001), lateral incisors (*R* = −0.583, *p* ~ 0.001), and canines (*R* = −0.386, *p* ~ 0.001) (Table 5).

**Table 5:** Correlations between the facial bone thickness and the (sagittal angulation, buccal bone concavity angle, and CEJ–BC distance) of maxillary anterior teeth.

Facial bone thickness										
		Central incisors			Lateral incisors			Canines		
		Coronal	Midroot	Apical	Coronal	Midroot	Apical	Coronal	Midroot	Apical
Sagittal angulation	*R*	−0.096	−0.177	−0.426	0.073	−0.081	−0.583	−0.034	−0.231	−0.386
	*p*‐Value	0.205	0.018 ^∗^	~0.001 ^∗∗^	0.327	0.278	~0.001 ^∗∗^	0.687	0.006 ^∗∗^	~0.001 ^∗∗^
Buccal bone concavity angle	*R*	−0.118	−0.134	0.082	−0.037	−0.009	0.275	−0.154	0.076	0.187
	*p*‐Value	0.119	0.076	0.276	0.622	0.906	~0.001 ^∗∗^	0.068	0.367	0.026 ^∗^
CEJ–BC	*R*	−0.151	0.025	−0.218	−0.058	0.197	−0.158	−0.220	0.277	0.011
	*p*‐Value	0.045 ^∗^	0.737	0.003 ^∗∗^	0.436	0.007 ^∗∗^	0.032 ^∗^	0.008 ^∗∗^	0.001 ^∗∗^	0.901

*Note: R*: Pearson’s correlation coefficient. *p*‐Value: significance (two‐tailed).

^∗^Correlation is significant at the 0.05 level (two‐tailed).

^∗∗^Correlation is significant at the 0.01 level (two‐tailed).

#### 3.10.2. The Palatal Bone Dimension

All palatal measurements showed significant positive correlation with sagittal angulation at all levels (*p*  < 0.001) (Table 6).

**Table 6:** Correlations between the palatal bone thickness and the (sagittal angulation, buccal bone concavity angle, and CEJ–BC distance) of maxillary anterior teeth.

Palatal bone thickness											
		Central incisors			Lateral incisors			Canines			
		Coronal	Midroot	Apical	Coronal	Midroot	Apical	Coronal	Midroot	Apical	
Sagittal angulation	*R*	0.281	0.493	0.609	0.375	0.475	0.664	0.287	0.579	0.482	
	*p*‐Value	~0.001 ^∗∗^	~0.001 ^∗∗^	~0.001 ^∗∗^	~0.001 ^∗∗^	~0.001 ^∗∗^	~0.001 ^∗∗^	0.001 ^∗∗^	~0.001 ^∗∗^	~0.001 ^∗∗^	
Buccal bone concavity angle	*R*	0.085	0.047	0.203	0.180	0.186	0.243	‐ 0.045	0.056	0.124	
	*p*‐Value	0.260	0.533	0.007 ^∗∗^	0.015 ^∗^	0.012 ^∗^	0.001 ^∗∗^	0.592	0.511	0.141	
CEJ–BC	*R*	0.108	0.106	0.162	−0.178	−0.128	−0.113	0.215	0.026	−0.067	
	*p*‐Value	0.151	0.160	0.032 ^∗^	0.016 ^∗^	0.083	0.127	0.010 ^∗^	0.759	0.429	

*Note: R*: Pearson’s correlation coefficient. *p*‐Value: significance (two‐tailed).

^∗^Correlation is significant at the 0.05 level (two‐tailed).

^∗∗^Correlation is significant at the 0.01 level (two‐tailed).

#### 3.10.3. The Distance Between CEJ and BC

The CEJ–BC distance of central incisors had significant positive correlation with the sagittal angle (*R* = 0.303, *p* ~ 0.001) (Table 7).

**Table 7:** Correlations between the sagittal angulation, buccal concavity angle, CEJ–BC distance of central, lateral incisors and canines.

		CEJ–BC								Buccal bone concavity angle			
		Central incisors				Lateral incisors	Canines			Central incisors		Lateral incisors	Canines
Sagittal angulation	*R*	0.303				0.104	0.013			0.166		0.082	−0.086
	*p*‐Value	~0.001 ^∗∗^				0.160	0.881			0.027 ^∗^		0.272	0.308
Buccal bone concavity angle	*R*	0.044				−0.047	−0.090			1		1	1
	*p*‐Value	0.565				0.527	0.285			—		—	—

*Note:* CEJ–BC: distance between CEJ and alveolar bone crest; *R*: Pearson’s correlation coefficient; *p*‐value: significance (two‐tailed).

^∗^Correlation is significant at the 0.05 level (two‐tailed).

^∗∗^Correlation is significant at the 0.01 level (two‐tailed).

#### 3.10.4. The Buccal Concavity Angle

The buccal concavity angle of central incisors showed a weak positive correlation with the sagittal angle (*R* = 0.166, *p* = 0.027) (Table 7) but when the correlations between the groups of angles of both the buccal concavity and the sagittal were investigated, no significant correlations were found (Table 8).

**Table 8:** Differences in the sagittal angulation of maxillary anterior teeth among different groups of the bone concavity angle using one‐way ANOVA (Mean ± SD).

Buccal concavity angle groups	Central incisors	*N*	Lateral incisors	*N*	Canines	*N*
115°–130°	8.86 ± 5.93	7	4.50 ± 3.50	2	—	0
131°–145°	8.04 ± 4.83	45	7.61 ± 4.71	41	11.77 ± 4.97	13
146°–160°	8.42 ± 4.84	71	7.63 ± 5.15	109	12.65 ± 4.53	69
161°–175°	8.27 ± 5.33	29	9.55 ± 5.76	29	11.82 ± 3.87	58
176°–190°	11.12 ± 5.45	25	4.50 ± 0.70	2	12.50 ± 0.70	2
Total	8.70 ± 5.09	177	7.86 ± 5.15	183	12.23 ± 4.27	142
*p*‐Value	0.146	—	0.275	—	0.722	—

*Note: p*‐Value: significant differences of one‐way ANOVA between groups.

### 3.11. Correlations Involving the Buccal Concavity Angle

#### 3.11.1. Facial Bone Dimensions

Apical facial bone thickness of lateral incisors and canines showed a significant positive correlation with the buccal concavity angle (*R* = 0.275, *p*  < 0.001; *R* = 0.187, *p* = 0.026, respectively) (Table 5).

#### 3.11.2. Palatal Bone Dimensions

Apical palatal bone thickness of central and lateral incisors demonstrated a significant positive correlation with the buccal concavity angle (*R* = 0.203, *p* = 0.007; *R* = 0.243, *p* = 0.001, respectively). In addition, palatal bone thickness at the coronal and midroot levels of lateral incisors also showed significant positive correlations (*R* = 0.180, *p* = 0.015; *R* = 0.186, *p* = 0.012, respectively) (Table 6).

#### 3.11.3. CEJ–BC Distance

No significant correlation was observed between CEJ–BC distance and the buccal concavity angle for maxillary anterior teeth (*p*  > 0.05) (Table 7).

#### 3.11.4. The Facial Bone Dimension

The thickness of the facial bone wall had a negative significant correlation with the CEJ–BC distance at coronal level of central incisors and canines (*R* = −0.151, *p* = 0.045; *R* = −0.220, *p* = 0.008), respectively, and at apical level of central and lateral incisors (*R* = −0.218, *p* = 0.003; *R* = −0.158, *p* = 0.032), respectively. While a positive correlation was found at midroot level of lateral incisors (*R* = 0.197, *p* = 0.007) and canines (*R* = 0.277, *p* = 0.001) (Table 5).

### 3.12. Group Comparisons (ANOVA)

Sagittal angulation was compared across groups of buccal concavity angle.

#### 3.12.1. Central Incisors

• No significant difference among concavity groups (*p* = 0.146).

#### 3.12.2. Lateral Incisors

• No significant difference (*p* = 0.275).

#### 3.12.3. Canines

• No significant difference (*p* = 0.722).

Although concavity categories varied widely, sagittal angulation did not change significantly across groups (Table 8).

### 3.13. Sensitivity Analysis Accounting for Within‐Patient Clustering (GEE)

The associations between sagittal angulation and both facial and palatal bone thickness (midroot and apical levels for facial thickness; all levels for palatal thickness), as well as between sagittal angulation and CEJ–BC distance of central incisors, remained statistically significant after clustering adjustment, supporting the robustness of these findings. Similarly, the gender differences in palatal bone thickness (all levels and all tooth types), CEJ–BC distance (central incisors and canines), and sagittal angulation (central incisors) remained significant. In contrast, several weaker associations did not remain significant after accounting for clustering, including most correlations between the buccal concavity angle and facial or palatal bone thickness (with the exception of lateral incisor apical facial thickness, which remained significant), most correlations between CEJ–BC distance and facial bone thickness, and the gender difference in lateral incisor facial thickness at the apical level. These findings suggest that the principal associations reported in this study are robust to within‐patient clustering, whereas some of the weaker secondary correlations should be interpreted with additional caution.

## 4. Discussion

In this study, CBCT was used to assess facial and palatal alveolar bone thickness, the distance between the CEJ and the facial BC, sagittal angulation, and buccal bone concavity angle in the anterior maxilla. While several previous studies have evaluated individual parameters, facial and palatal bone dimensions [7, 9, 17], CEJ–BC distance [8, 9, 18], sagittal angulation [12, 13], and buccal bone concavity angle [9, 10]. To our knowledge, no study has simultaneously evaluated all these parameters and explored their interrelationships, providing a more integrated understanding of anterior maxillary anatomy relevant to implant planning. Sagittal angulation is particularly critical for dental implant placement. Ideally, the implant axis should align with the natural tooth axis and be positioned slightly more palatally. However, discrepancies between the tooth axis and optimal implant trajectory often necessitate angled osteotomies, guided surgery, or bone augmentation. In the present study, canines exhibited the greatest sagittal angulation among anterior teeth, followed by central incisors and then lateral incisors. This pattern reflects the functional prominence of canines and their position along the curvature of the dental arch. These findings are consistent with previous reports [12, 13, 19]. Lopez‐Jarana et al. [12] assessed 99 maxillary anterior teeth, reporting mean sagittal angles of 11.67° ± 6.4° for incisors and 16.88° ± 7.9° for canines, while dos Santos et al. [19] found mean values of 12.7° ± 5.4°, 14.8° ± 6.5°, and 16.5° ± 7.2° for central incisors, lateral incisors, and canines, respectively. The pronounced angulation of canines likely reflects their role in lateral guidance and their prominent root morphology.

Sagittal angulation of ≤10° was observed in 66.1% of maxillary central incisors, 69.5% of lateral incisors, and 35.9% of canines. These percentages are slightly different than those in the literature [12, 13, 19]. Lopez‐Jarana et al. [12] observed that 47% of central incisors, 53% of lateral incisors, and 18% of canines had angles less than 10°. In another study, dos Santos et al. [19] found that 35.9% of central incisors, 22.5% of lateral incisors, and 13.5% of canines had an angle less than 10°. Other studies reported small percentage of teeth with sagittal angulations of less than 10°. For instance, Wang et al. [13] reported that only 10% of maxillary anterior teeth had tooth angles <10°. The percentages of teeth with angulation ≤10° were similar but not identical to values in previous studies. The observed disparities may relate to differences in imaging systems, voxel resolution, or methodological definitions of the alveolar ridge axis.

In the present study, canines showed less concave buccal plate compared to central incisors, as shown by the buccal bone concavity angles. This might be explained by the proclination of anterior maxilla that is necessary to enable the incisors to overlap mandibular anterior teeth and the presence of the lateral fossa around the apices of lateral incisors. Furthermore, the presence of canine eminence on the labial bone wall overlying the canine at the corner of the arch had a cosmetic value in maintaining the harmony for normal facial expressions. These findings are similar to the results of previous studies. Lee et al. [9] found that central incisors have the most curved buccal bone wall. Chung et al. [10] studied the location and depth of buccal concavity in anterior maxilla in 11 cadaver heads, reporting the deepest buccal concavity at lateral incisors using CBCT and direct measurements.

The results of the present study show that the thin facial bone wall (<1.5 mm) [20] predominates in the anterior maxilla with a rare incidence of a thick bone wall. This might indicate that facial bone thickness could be genetically determined, especially that similar findings were observed in people with different ethnic origins [7, 9, 17]. A recent systematic review and meta‐analysis included 29 studies that measured the facial bone thickness of maxillary anterior teeth using the CBCT, reporting the mean thickness to be 1 mm or less and varied between 0.75 and 1.05 mm [21]. In a CBCT‐based study of 20 Korean subjects, Lee et al. [9] analyzed the facial and palatal bone thicknesses of maxillary anterior teeth at different levels (coronal, middle, and apical), reporting that facial bone thickness was less than 1 mm, while the palatal bone wall thickness varied between 1.40 ± 0.84 and 2.39 ± 1.27 mm coronally, between 1.83 ± 0.96 and 2.95 ± 1.63 mm at midroot, and between 3.81 ± 1.63 and 7.29 ± 2.90 mm apically. In another CBCT study, Gluckman et al. [17] measured the buccal and palatal bone thicknesses of 951 maxillary anterior teeth at crestal, middle, and apical levels, reporting the facial bone thickness were 0.6 ± 0.3, 0.5 ± 0.3, and 1.2 ± 0.8 mm, respectively, while those of the palatal bone thickness were 1.0 ± 0.7, 3.8 ± 1.6, and 7.7 ± 2.9 mm. Furthermore, AlTarawneh et al.[7] observed that more than 80% of maxillary anterior teeth had thin buccal bone thickness (<1 mm), while the mean thickness of the palatal bone wall for maxillary anterior teeth varied between 0.87 and 1.10 mm coronally and between 1.52 and 1.75 mm at midroot, while it varied between 2.66 and 3.60 mm apically. In addition, the palatal bone is significantly thicker than buccal bone [7, 9, 17]. Similarly, the markedly thicker palatal bone observed in this study, particularly apically, may reinforce the clinical practice of favoring palatal implant positioning whenever feasible.

The thickness of the midroot bone level was very thin compared to the coronal and apical levels, which may render this region particularly susceptible to dehiscence or perforation during osteotomy preparation, especially in lateral incisors and canines. This finding resembles the “hourglass” alveolar morphology described by Ahmed and Elbeshlawy [22].

In this study, the distance between the CEJ and the facial BC of maxillary anterior teeth averaged between 1.65 ± 0.56 and 1.72 ± 0.56, wherein 60% of the teeth had a distance varied between 1 and 2 mm, while 30% had a distance between 2 and 3 mm. Normal distance of the CEJ–BC was in a range between 1 and 3 mm to accommodate the supracrestal tissue attachment and maintain a stable gingival tissue around teeth. This finding is consistent with those reported in previous studies [8, 9, 13, 18]. A recent systematic review and meta‐analysis reported the means of the CEJ–BC distance to vary between 2 and 2.5 mm [21]. Nevertheless, few previous studies had observed wide range of the CEJ–BC distance of greater than 3 mm [5, 23]. The disparity in the measurements of the CEJ–BC distance between the studies could be attributed to different inclusion criteria, in which subjects with crestal bone loss had been included. For instance, Ghassemian et al. [23] observed a wide range for the distance of CEJ–BC in maxillary anterior teeth varied from 0.8 to 7.2 mm and reported greater distance of CEJ–BC for smokers and patients of 50 years or older. In another study, Nowzari et al. [5] observed that 3.5% of the maxillary central incisors had a CEJ–BC distance of greater than or equal to 4 mm and found significant differences between African Americans and Hispanic Americans.

### 4.1. Correlations Between the Measurements

In the present study, a weak significant correlation was found between the facial bone thickness and the distance of CEJ–BC of maxillary anterior teeth, in which the measurements of central incisors at coronal and apical levels, lateral incisors at apical levels, and canines at coronal levels displayed tendency to decrease as the distance increase. On the other side, the midroot measurements of the facial bone of lateral incisors and canines showed a tendency of increase as the distance of CEJ–BC increase. This finding might be attributed to the continuous tooth eruption and the upright tooth movement that occurs throughout life and which adjoined with remodeling processes of resorption and deposition of alveolar bone [24].

Furthermore, an inverse correlation was observed between sagittal angulation and apical facial bone thickness, suggesting that greater tooth inclination is associated with a thinner facial plate, which may increase the risk of cortical compromise during implant placement. Other studies [15, 16] reported similar findings. Conversely, palatal bone thickness exhibited a strong positive correlation with sagittal angulation; these findings support the prominent root position of the maxillary anterior teeth.

The results also showed that the buccal bone concavity angle tends to increase (i.e., become less concave) as apical facial bone thickness of lateral incisors and canines increases, suggesting that the buccal concavity and sagittal angulation, consistent with the findings of Botermans et al. [14].

Because multiple teeth from the same patient were included in the analyses above, all gender comparisons and correlation analyses were reassessed using GEE with an exchangeable working correlation structure, clustering observations by patient (Supporting Information 2: Table S1, available as supporting file). This sensitivity analysis confirmed that the principal findings of this study are robust to within‐patient clustering: the correlations between sagittal angulation and both facial bone thickness (midroot and apical levels) and palatal bone thickness (all levels), the correlation between sagittal angulation and CEJ–BC distance of central incisors, and the main gender differences (palatal bone thickness at all levels, CEJ–BC distance in central incisors and canines, and sagittal angulation in central incisors) all remained statistically significant after clustering adjustment. In contrast, several weaker, secondary associations did not remain significant once clustering was accounted for, including most correlations between the buccal concavity angle and facial or palatal bone thickness (with the exception of lateral incisor apical facial thickness, which remained significant), most correlations between CEJ–BC distance and facial bone thickness (with the exception of the central incisor coronal‐level and canine midroot correlations), and the gender difference in lateral incisor facial thickness at the apical level. These weaker associations should therefore be interpreted as preliminary rather than robust findings, whereas the principal associations underlying the clinical implications discussed below can be regarded as robust to within‐patient clustering.

There are some limitations in the present study. First, CBCT has limited accuracy in detecting ultrathin cortical plates (<0.4 mm), which may lead to underestimation of bone presence. Second, the older age subgroup (51–75 years) was underrepresented, limiting assessment of age‐related remodeling trends. Third, although correlations were identified, the cross‐sectional design precludes causal interpretation. Fourth, as multiple teeth from the same patient were included, observations were not fully statistically independent. Fifth, this study evaluated only hard‐tissue parameters; soft‐tissue characteristics (e.g., gingival phenotype, keratinized tissue width, and mucosal thickness), which also influence implant esthetics and augmentation needs, were not assessed and should be incorporated in future research.

### 4.2. Clinical Implications

The findings of this study have several important implications for clinicians planning implant placement in the anterior maxilla.

#### 4.2.1. Anticipation of Thin Facial Bone

Since the majority of anterior maxillary teeth exhibited a thin facial bone wall (<1.5 mm), clinicians should expect a high risk of buccal plate resorption following extraction or implant placement. This reinforces the importance of atraumatic extraction techniques and the frequent need for bone augmentation or immediate grafting procedures to preserve the buccal contour and avoid midfacial recession. Importantly, these findings suggest that immediate implant placement should not be the default approach in all anterior cases. In sites with extremely thin buccal bone, a delayed or staged protocol may provide a more predictable outcome in terms of hard and soft tissue stability.

#### 4.2.2. Implant Positioning Challenges: Angulation, Concavity, and Midroot Defects

The anterior maxilla often presents a combination of factors that complicate implant positioning. Discrepancies between the natural tooth axis and the ideal implant trajectory—particularly in canines with greater sagittal angulation—can require palatal positioning, angled abutments, or guided surgery. Central incisors frequently exhibit deeper buccal concavities, heightening the risk of cortical plate perforation during osteotomy preparation. Furthermore, the study revealed that the midroot region is especially thin, predisposing lateral incisors and canines to dehiscence or fenestration. Together, these findings highlight the necessity of detailed CBCT‐based evaluation, cautious drilling, and frequent consideration of ridge preservation or augmentation procedures to achieve stable and esthetic results.

#### 4.2.3. Gender and Tooth‐Specific Variations

Since men exhibited significantly greater bone dimensions and angulations than women, and given the differences observed between tooth types, clinicians should individualize treatment planning according to both patient demographics and tooth site. Most importantly, clinicians should avoid a “one‐size‐fits‐all” approach to immediate implant placement and instead tailor timing and technique to the patient’s anatomy to minimize complications and optimize esthetic outcomes.

## 5. Conclusion

The present study demonstrated that the anterior maxilla is characterized by consistently thin facial bone and substantially thicker palatal bone, a CEJ–BC distance within the physiologic range, and pronounced sagittal inclination, especially in canines. Men showed significantly greater measurements than women, particularly of facial and palatal bone thicknesses, distance of CEJ–BC (central incisors and canines), and sagittal angulation (central incisors). Significant correlations were identified among bone thickness, angulation, and CEJ–BC distance, offering insight into anatomical patterns relevant to implant placement.

Certain parameter pairs demonstrated no meaningful correlation, indicating that not all anatomical features co‐vary. Further studies with larger and more age‐diverse samples are needed to expand the understanding of hard‐ and soft‐tissue relationships and their influence on implant esthetics.

## Funding

No funding was received for this study.

## Conflicts of Interest

The authors declare no conflicts of interest.

## Supporting Information

Additional supporting information can be found online in the Supporting Information section.

## Supporting information


**Supporting Information 1** Figure S1: Flow diagram of patient and tooth selection, prepared in accordance with STROBE guidelines. More than 1000 scans were assessed for eligibility, of which 112 (33 men, 79 women; mean age 31.10 ± 10.67 years, range 18–71) met the inclusion criteria and were included. Across these 112 patients, 672 maxillary anterior teeth (six per patient: central incisors, lateral incisors, and canines, bilaterally) were identified; 170 teeth were subsequently excluded (crestal or periapical bone loss, tooth surface loss, malalignment, full‐coverage restorations or veneers, incisal restorations, endodontic treatment, root resorption, or motion/metal artifacts), leaving a final sample of 502 teeth (177 central incisors, 184 lateral incisors, 142 canines) for analysis.

**Supporting Information 2** Table S1: Sensitivity analysis using generalized estimating equations (GEE) accounting for within‐patient clustering. Gender comparisons and correlation analyses from the primary analysis (independent *t*‐tests and Pearson correlations, which treat each tooth as an independent observation) were reassessed using GEE with an exchangeable working correlation structure, clustering observations by patient and using robust (sandwich) standard errors. Rows shaded pink indicate an association that was statistically significant in the primary (naive) analysis but did not remain significant after adjustment for within‐patient clustering.

## Data Availability

The data that support the findings of this study are available from the corresponding author upon reasonable request.
