# Supplementary material for: Cone‐Beam Computed Tomography–Derived Alveolar Anatomy for Immediate Implant Planning: Thickness, Concavity, and Angulation Patterns: A Comprehensive Evaluation
Source: Int J Dent. 2026 Sep 17;2026:9240109. doi: 10.1155/ijod/9240109 (PMC13586788; doi:10.1155/ijod/9240109)
Supplement: Supplementary file 1 [file IJOD-2026-9240109-s001.docx]

**Table S1. Sensitivity analysis using Generalized Estimating Equations (GEE) accounting for within-patient clustering**

Gender comparisons and correlation analyses from the primary analysis (independent t-tests and Pearson correlations, which treat each tooth as an independent observation) were re-assessed using GEE with an exchangeable working correlation structure, clustering observations by patient and using robust (sandwich) standard errors. Rows shaded pink indicate an association that was statistically significant in the primary (naive) analysis but did not remain significant after adjustment for within-patient clustering.

**Table S1a. Gender comparisons**

| **Tooth type** | **Outcome** | **n** | **Male (mean ± SD)** | **Female (mean ± SD)** | **p (naive t-test)** | **p (GEE, clustered)** |
| --- | --- | --- | --- | --- | --- | --- |
| Central | Facial Coronal | 177 | 1.04 ± 0.28 | 1.06 ± 0.25 | 0.641 | 0.680 |
| Central | Facial Mid | 177 | 0.79 ± 0.45 | 0.77 ± 0.34 | 0.722 | 0.885 |
| Central | Facial Apical | 177 | 1.41 ± 0.79 | 1.30 ± 0.76 | 0.395 | 0.494 |
| Central | Palatal Coronal | 177 | 2.17 ± 0.59 | 1.83 ± 0.53 | <0.001 | 0.002 |
| Central | Palatal Mid | 177 | 3.77 ± 1.14 | 2.93 ± 0.95 | <0.001 | <0.001 |
| Central | Palatal Apical | 177 | 8.70 ± 2.22 | 7.12 ± 1.92 | <0.001 | <0.001 |
| Central | CEJ BC | 177 | 1.88 ± 0.57 | 1.57 ± 0.52 | 0.001 | 0.005 |
| Central | Sagittal Angle | 177 | 10.84 ± 5.52 | 8.27 ± 4.65 | 0.004 | 0.012 |
| Central | Concavity Angle | 177 | 150.91 ± 19.53 | 151.68 ± 14.14 | 0.796 | 0.912 |
| Lateral | Facial Coronal | 184 | 1.16 ± 0.39 | 1.21 ± 0.51 | 0.487 | 0.588 |
| Lateral | Facial Mid | 184 | 0.61 ± 0.59 | 0.72 ± 0.66 | 0.281 | 0.312 |
| Lateral | Facial Apical | 184 | 1.91 ± 1.14 | 1.43 ± 1.10 | 0.009 | 0.053 |
| Lateral | Palatal Coronal | 184 | 1.82 ± 0.60 | 1.42 ± 0.57 | <0.001 | <0.001 |
| Lateral | Palatal Mid | 184 | 3.22 ± 1.21 | 2.21 ± 1.01 | <0.001 | <0.001 |
| Lateral | Palatal Apical | 184 | 6.90 ± 1.96 | 5.55 ± 1.92 | <0.001 | 0.001 |
| Lateral | CEJ BC | 184 | 1.77 ± 0.53 | 1.71 ± 0.57 | 0.481 | 0.569 |
| Lateral | Sagittal Angle | 184 | 7.93 ± 5.00 | 8.06 ± 5.73 | 0.873 | 0.908 |
| Lateral | Concavity Angle | 184 | 154.12 ± 8.12 | 151.80 ± 9.76 | 0.096 | 0.138 |
| Canine | Facial Coronal | 142 | 1.03 ± 0.42 | 1.14 ± 0.35 | 0.195 | 0.350 |
| Canine | Facial Mid | 142 | 0.79 ± 0.56 | 0.48 ± 0.51 | 0.007 | 0.025 |
| Canine | Facial Apical | 142 | 1.63 ± 1.07 | 1.05 ± 0.92 | 0.006 | 0.007 |
| Canine | Palatal Coronal | 142 | 1.87 ± 0.63 | 1.39 ± 0.45 | <0.001 | <0.001 |
| Canine | Palatal Mid | 142 | 4.55 ± 1.93 | 3.15 ± 1.26 | <0.001 | 0.001 |
| Canine | Palatal Apical | 142 | 10.92 ± 3.76 | 8.89 ± 2.66 | 0.005 | 0.035 |
| Canine | CEJ BC | 142 | 2.09 ± 0.50 | 1.51 ± 0.51 | <0.001 | <0.001 |
| Canine | Sagittal Angle | 142 | 13.29 ± 4.93 | 12.14 ± 4.52 | 0.230 | 0.397 |
| Canine | Concavity Angle | 142 | 160.00 ± 7.22 | 157.51 ± 7.85 | 0.091 | 0.145 |

**Table S1b. Correlation analyses**

| **Tooth type** | **Association** | **n** | **Pearson r** | **p (naive)** | **p (GEE, clustered)** |
| --- | --- | --- | --- | --- | --- |
| Central | SagAngle vs Facial-Coronal | 177 | -0.096 | 0.204 | 0.264 |
| Central | SagAngle vs Facial-Mid | 177 | -0.181 | 0.016 | 0.030 |
| Central | SagAngle vs Facial-Apical | 177 | -0.428 | <0.001 | <0.001 |
| Lateral | SagAngle vs Facial-Coronal | 184 | 0.080 | 0.282 | 0.393 |
| Lateral | SagAngle vs Facial-Apical | 184 | -0.574 | <0.001 | <0.001 |
| Canine | SagAngle vs Facial-Mid | 142 | -0.251 | 0.003 | 0.005 |
| Canine | SagAngle vs Facial-Apical | 142 | -0.394 | <0.001 | <0.001 |
| Central | SagAngle vs Palatal-Coronal | 177 | 0.278 | <0.001 | 0.005 |
| Central | SagAngle vs Palatal-Mid | 177 | 0.493 | <0.001 | <0.001 |
| Central | SagAngle vs Palatal-Apical | 177 | 0.609 | <0.001 | <0.001 |
| Lateral | SagAngle vs Palatal-Coronal | 184 | 0.352 | <0.001 | <0.001 |
| Lateral | SagAngle vs Palatal-Mid | 184 | 0.440 | <0.001 | <0.001 |
| Lateral | SagAngle vs Palatal-Apical | 184 | 0.637 | <0.001 | <0.001 |
| Canine | SagAngle vs Palatal-Coronal | 142 | 0.259 | 0.002 | 0.017 |
| Canine | SagAngle vs Palatal-Mid | 142 | 0.556 | <0.001 | <0.001 |
| Canine | SagAngle vs Palatal-Apical | 142 | 0.505 | <0.001 | <0.001 |
| Central | SagAngle vs CEJ-BC | 177 | 0.306 | <0.001 | 0.001 |
| Central | SagAngle vs ConcavityAngle | 177 | 0.044 | 0.562 | 0.904 |
| Lateral | ConcavityAngle vs Facial-Apical | 184 | 0.275 | <0.001 | 0.015 |
| Canine | ConcavityAngle vs Facial-Apical | 142 | 0.174 | 0.038 | 0.146 |
| Central | ConcavityAngle vs Palatal-Apical | 177 | 0.086 | 0.258 | 0.657 |
| Lateral | ConcavityAngle vs Palatal-Apical | 184 | 0.245 | <0.001 | 0.113 |
| Lateral | ConcavityAngle vs Palatal-Coronal | 184 | 0.180 | 0.015 | 0.178 |
| Lateral | ConcavityAngle vs Palatal-Mid | 184 | 0.185 | 0.012 | 0.167 |
| Central | ConcavityAngle vs CEJ-BC | 177 | -0.018 | 0.809 | 0.976 |
| Lateral | ConcavityAngle vs CEJ-BC | 184 | -0.048 | 0.517 | 0.562 |
| Canine | ConcavityAngle vs CEJ-BC | 142 | -0.090 | 0.285 | 0.466 |
| Central | CEJ-BC vs Facial-Coronal | 177 | -0.151 | 0.045 | 0.032 |
| Canine | CEJ-BC vs Facial-Coronal | 142 | -0.220 | 0.009 | 0.132 |
| Central | CEJ-BC vs Facial-Apical | 177 | -0.218 | 0.004 | 0.379 |
| Lateral | CEJ-BC vs Facial-Apical | 184 | -0.159 | 0.032 | 0.091 |
| Lateral | CEJ-BC vs Facial-Mid | 184 | 0.195 | 0.008 | 0.260 |
| Canine | CEJ-BC vs Facial-Mid | 142 | 0.277 | <0.001 | 0.008 |

*Facial = buccal/facial bone thickness; Palatal = palatal bone thickness; CEJ-BC = distance between cemento-enamel junction and facial bone crest; SagAngle = sagittal angulation; ConcavityAngle = buccal bone concavity angle. GEE models used an exchangeable working correlation structure with Patient_ID as the clustering variable and robust standard errors. Naive p-values replicate the original independent-samples t-test (gender comparisons) or Pearson correlation (association analyses), which do not account for multiple teeth being measured within the same patient.*
